# Retraction Statement: Personality traits that associate with sustainable behaviors perceived by individuals

**DOI:** 10.1002/brb3.3393

**Published:** 2024-01-11

Longlong, Z. (2023). Personality traits that associate with sustainable behaviors perceived by individuals. *Brain and Behavior*, 13, e3147. https://doi.org/10.1002/brb3.3147

The above article, published online on July 11, 2023, in Wiley Online Library (wileyonlinelibrary.com), has been retracted by agreement between the author, the journal Editor‐in‐Chief Amanda Bischoff‐Grethe, and Wiley Periodicals, LLC. The retraction has been agreed due to concerns raised by a third party regarding significant errors in the descriptive statistics and intercorrelations for the HEXACO‐60 scales, reported in Tables 1 and 2. The author was unable to provide compelling raw data or a satisfactory response. Therefore, the data and the conclusions of the manuscript can no longer be considered reliable.

